# Molecular Pathology of Ovarian Endometrioid Carcinoma: A Review

**DOI:** 10.32604/or.2025.068432

**Published:** 2025-11-27

**Authors:** Hiroshi Yoshida, Mayumi Kobayashi Kato

**Affiliations:** 1 Department of Diagnostic Pathology, National Cancer Center Hospital, 5-1-1 Tsukiji, Chuo-ku, Tokyo, 104-0045, Japan; 2 Department of Gynecology, National Cancer Center Hospital, 5-1-1 Tsukiji, Chuo-ku, Tokyo, 104-0045, Japan

**Keywords:** Ovarian cancer, endometrioid carcinoma, diagnosis, pathology, immunohistochemistry, genomic analysis

## Abstract

Ovarian endometrioid carcinoma (OEC) accounts for ~10% of epithelial ovarian cancers and displays broad morphologic diversity that complicates diagnosis and grading. Recent data show that the endometrial cancer molecular taxonomy (DNA polymerase epsilon, catalytic subunit [POLE]-ultramutated, mismatch repair-deficient [MMRd], p53-abnormal, no specific molecular profile [NSMP]) also applies to OEC, and that OEC is enriched for Lynch syndrome–associated tumors, supporting routine MMR testing. We aimed to synthesize contemporary evidence spanning epidemiology, histopathology and immunophenotype, diagnostic pitfalls and differential diagnosis, and to evaluate the clinical utility of The Cancer Genome Atlas (TCGA)-surrogate molecular classification for risk stratification; we also summarize implications for Lynch screening, genetic counseling, and therapeutic opportunities including immune checkpoint inhibitors and targeted approaches, with practical recommendations for diagnostic workflows. Integrating morphology with molecular classification refines diagnosis and prognostication: POLEmut/MMRd subsets generally have excellent outcomes and are candidates for de-escalation or immunotherapy, whereas p53abn/high-grade tumors carry a poorer prognosis and may warrant intensified management and trials of homologous recombination deficiency (HRD)-directed strategies; routine MMR immunohistochemistry (IHC) with reflex germline testing improves Lynch detection, and future priorities include prospective validation and multi-omics to refine NSMP and identify new targets.

## 1 Introduction

Ovarian cancer remains a significant global health burden, with an estimated 314,000 new cases and 207,000 deaths reported worldwide in 2020, ranking as the eighth leading cause of cancer-related mortality among women [1,2]. Among the diverse histologic subtypes of epithelial ovarian carcinoma, ovarian endometrioid carcinoma (OEC) accounts for approximately 10% of cases [3–6]. According to the 2020 World Health Organization (WHO) classification, OEC is defined as a carcinoma resembling endometrioid carcinoma of the uterine corpus, characterized by tubular glands resembling proliferative endometrial epithelium [7]. However, OEC encompasses a broad morphological spectrum, often including glandular, solid, squamous, or sertoliform patterns, which may lead to diagnostic confusion with other primary ovarian neoplasms or metastatic tumors [8]. With the advancement of high-throughput sequencing technologies, comprehensive genomic analyses have unveiled recurrent molecular alterations in OEC and have enabled the application of molecular classification models—originally developed for endometrial carcinoma—to ovarian tumors [9–11]. This review aims to provide an up-to-date overview of OEC, focusing on general pathological considerations, tumorigenesis, diagnostic pitfalls, and recent insights into molecular alterations, emphasizing their clinical and biological significance.

## 2 Epidemiology and Risk Factors of OEC

OEC accounts for approximately 10% of all ovarian carcinomas [3–6]. While earlier studies reported a higher frequency of OEC and a lower incidence of high-grade serous carcinoma (HGSC), this discrepancy likely reflects historical misclassification, particularly of SET (Solid, pseudo-Endometrioid, and Transitional cell-like)-pattern HGSCs as high-grade OECs [12,13]. Recent studies employing refined pathological classification, including appropriate immunohistochemistry, consistently report the frequency of OEC as around 10%, with no significant difference between Western and Asian populations [4]. This contrasts with ovarian clear cell carcinoma (OCCC), which is more prevalent among Asian women [4].

Endometriosis is a well-established risk factor for OEC, conferring a two- to threefold increase in risk, a feature shared with OCCC [4,14–16]. Additional risk factors include hormonal and reproductive factors, such as elevated estrogen exposure and a greater number of lifetime menstrual cycles, which are associated with an increased risk [14,17]. Obesity, with a 1.2-fold increase in risk per 5 kg/m² increase in body mass index (BMI), and hormone replacement therapy (relative risk ~1.3–1.4) are also associated with a higher OEC risk [4,14,18]. In contrast, factors that reduce menstrual frequency—such as higher parity (with each birth reducing the risk by ~10%) and oral contraceptive use (a 15%–20% risk reduction with five years of use)—are protective [14,19]. Surgical interventions like tubal ligation and salpingectomy also appear protective [14,20], possibly by reducing retrograde menstruation. Interestingly, unlike other ovarian cancer subtypes, smoking was correlated with a lower risk for OEC (relative risk ~0.8) [21,22]. Although OEC and OCCC share many risk factors due to their association with endometriosis, OEC shows stronger associations with a family history of ovarian cancer and higher BMI, features not typically observed in OCCC [4].

In Japan, OEC is increasingly diagnosed in younger women (<50 years) and in early-stage disease [23]. This trend may reflect the rising prevalence of endometriosis, increased lifetime menstrual cycles due to declining fertility, delayed marriage, earlier menarche, and later menopause, as well as the growing prevalence of obesity. These factors are thought to be influenced by recent societal and lifestyle changes that impact reproductive and hormonal health.

## 3 Carcinogenesis of OEC: Association with Endometriosis

Endometriosis is a well-established epidemiological risk factor for OEC, with histological evidence of endometriosis observed in approximately 40% of resected OEC specimens [24,25]. The presence of shared somatic mutations between endometriotic lesions and OEC—such as AT-rich interaction domain 1A *(ARID1A*) loss-of-function and phosphatidylinositol-4,5-bisphosphate 3-kinase catalytic subunit alpha (*PIK3CA*) gain-of-function mutations—supports the hypothesis that OEC may arise from endometriosis [26,27]. This pathogenetic model has practical implications for diagnostic pathology, particularly in interpreting endometrioid or clear cell carcinomas at extra-gynecologic sites (e.g., abdominal wall, inguinal region, vulva) [28].

Endometriosis affects 10%–18% of reproductive-aged women and is associated with pelvic pain, infertility, and a 2%–3% lifetime risk of ovarian cancer, exceeding that of the general female population [29,30]. However, predictive markers to identify which endometriosis patients are at highest risk for malignant transformation remain unknown. Identifying such markers could enable targeted surveillance or preventive surgery. Notably, even morphologically benign endometriotic lesions have been shown to harbor oncogenic mutations (e.g., *ARID1A, PIK3CA, KRAS, PTEN, TP53*), indicating their potential as precursors to malignancy [31–33].

A large-scale genome-wide association study meta-analysis, combined with Mendelian randomization, provided genetic evidence supporting a causal direction from endometriosis to specific histotypes of epithelial ovarian carcinoma [34]. This study identified shared genetic loci, candidate functional genes, and signaling pathways linking endometriosis and histotype-specific ovarian cancer risk [34]. Despite their frequent co-occurrence with endometriosis, OEC and OCCC differ markedly in phenotype and clinical behavior; OCCC is chemoresistant and more aggressive. In contrast, OEC is typically of low grade with a favorable prognosis [3,12]. Intriguingly, OEC and OCCC share similar mutational profiles, including *ARID1A* and *PIK3CA* mutations [27,35], which are also present in benign endometriotic lesions, suggesting that these mutations alone do not dictate the histologic subtype.

Transcriptional and epigenetic differences likely underpin the divergence between OEC and OCCC [36]. Hepatocyte nuclear factor 1 Beta (HNF1β) is strongly expressed in OCCC and is involved in metabolic reprogramming (e.g., increased glycolysis and lactate secretion) [37]. Genetic polymorphisms in *HNF1Β* (*HNF1 homeobox B*) are protective against OEC but increase susceptibility to OCCC [38]. These findings suggest that OCCC and OEC arise from a common progenitor but become locked into distinct cellular states, reflecting different phases of the menstrual cycle. This model was supported by Beddows et al., who demonstrated via RNA and DNA methylation profiling that OEC and OCCC transcriptionally mimic proliferative and secretory endometrium, respectively [36]. These insights may reveal novel therapeutic targets, particularly for OCCC, which lacks effective treatments and remains a clinical challenge.

## 4 Clinical Presentation of OEC

OEC typically presents in perimenopausal women, with a median age at diagnosis ranging from 54 to 58 years [5,6]. Most cases are diagnosed at an early stage, with International Federation of Gynecology and Obstetrics (FIGO) stage I accounting for roughly 50%, stage II for 20%–40%, and stage III/IV for 10%–25% of cases [39,40]. Most tumors are low-grade, with grade 1 representing 50%–60%, grade 2 approximately 30%, and grade 3 around 20% [39,40]. Bilateral ovarian involvement is seen in 17% of patients, and coexisting endometrial carcinoma is present in 15%–20% of cases [7,41].

OEC commonly arises in association with endometriotic cysts and is often accompanied by components of endometrioid adenofibroma or borderline tumors. Endometriosis is identified in up to 42% of cases within the ipsilateral ovary or pelvic cavity [24,25]. Given their typically low-grade histology and confinement to the ovary, OECs generally have a favorable prognosis compared to other ovarian cancer histotypes [12,42]. However, a subset of cases with high-grade or advanced-stage disease is associated with poorer outcomes [42].

Radiologically, OEC and OCCC often appear as mixed solid and cystic masses, with a greater proportion of solid components and occasional hemorrhage, distinguishing them from serous or mucinous tumors [43]. When arising within endometriotic cysts, these tumors frequently manifest as mural nodules with vascularity or contrast enhancement on ultrasound or MRI—particularly in women over 45 years—raising suspicion for malignancy [44,45]. On MRI, non-hemorrhagic secretions produced by the tumor may dilute the cyst content, leading to loss of the T2 shading sign. While CT findings are nonspecific, high-attenuation areas may indicate intratumoral hemorrhage [44,45]. These findings support a correct radiological diagnosis of ovarian cancer.

## 5 Histopathological Findings of OEC

### 5.1 Confirmatory Endometrioid Features

**Figure 1:**
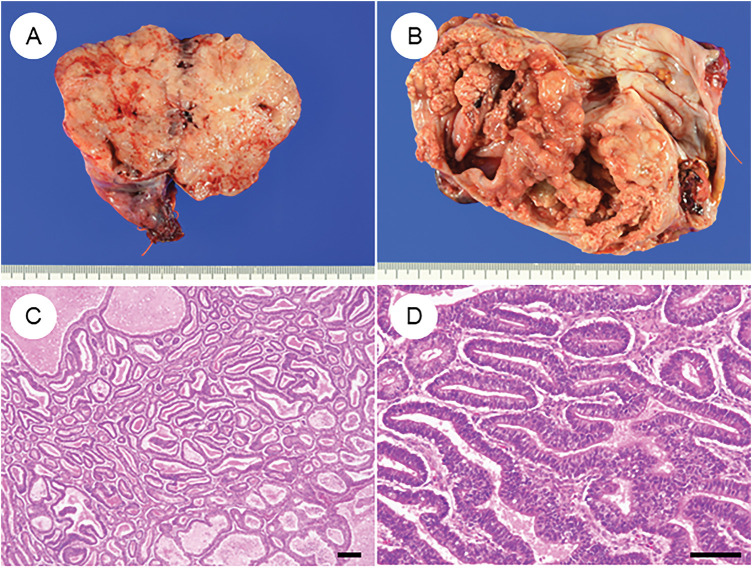

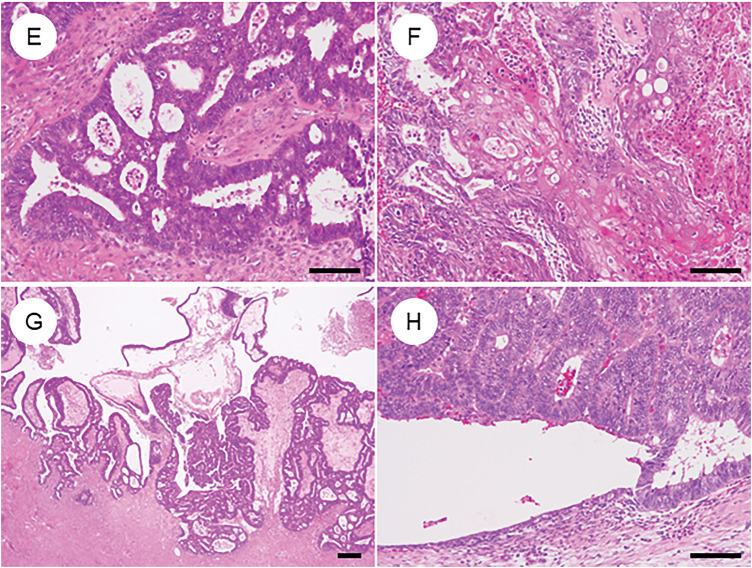
Characteristic histopathological features of ovarian endometrioid carcinoma. (**A**) Gross appearance of a tumor is predominantly composed of solid components. (**B**) Tumor with solid areas arising within a cystic space. (**C**) A dense proliferation of tumor glands in a “back-to-back” arrangement. (**D**) Tumor glands exhibit smooth luminal surfaces and pseudostratified nuclei. (**E**) Cribriform fusion of neoplastic glands. (**F**) Squamous differentiation. (**G**) Ovarian endometrioid carcinoma may be associated with a borderline tumor component at the periphery. (**H**) Endometriosis is occasionally observed adjacent to the tumor. The black bar represents 200 μm. All pathology images are original images obtained from the pathology image database of the National Cancer Center Hospital (C, G: ×40; D–F, H: ×200)

Macroscopically, OECs typically present as large (>10 cm) unilateral masses [41]. When arising from endometriotic cysts, they characteristically form mural nodules protruding into the cystic lumen. Tumors with an adenofibromatous background often appear solid and are more likely to exhibit hemorrhage and necrosis (Fig. 1A and B) [41].

Histologically, OECs resemble endometrioid carcinomas of the uterine corpus, displaying a spectrum of metaplastic and architectural changes [7,8,41]. The classic pattern features closely packed glands arranged in a back-to-back, cribriform, or labyrinthine structure. Tumor glands are lined by tall columnar cells with smooth luminal borders, round to oval nuclei, and frequent pseudostratification (Fig. 1C–E). Nuclear atypia is typically mild to moderate, although mitotic activity varies among cases. Solid growth areas may be present and are often associated with higher nuclear atypia and necrosis compared to glandular areas. In addition to these classic features, OECs commonly exhibit metaplastic changes, particularly squamous (Fig. 1F) and endocervical-type mucinous metaplasia. These “confirmatory endometrioid features” are essential diagnostic clues [41,46]. Recognition of morphological resemblance to typical endometrial endometrioid carcinoma, the presence of metaplasia, and identification of associated precursor lesions such as endometriosis, adenofibroma, or borderline tumors are all critical for accurate diagnosis and differential considerations (Fig. 1G and H). Establishing these features is especially important when distinguishing OEC from histologic mimics.

### 5.2 Tumor Grading (Fig. 2)

Due to morphological similarities, the FIGO grading system for endometrial carcinoma has traditionally been applied to OEC [7]. However, concerns have been raised regarding the lack of robust, evidence-based validation of FIGO grading in this context. In contrast, the Silverberg grading system, widely used for ovarian carcinoma, has demonstrated established associations with overall and disease-free survival [47,48]. Ishioka et al., analyzing a large ovarian cancer cohort, found both FIGO and Silverberg grading to be significant prognostic indicators. However, the Silverberg system strongly correlated with lymph node metastasis and residual disease, suggesting its potential superiority [49].

**Figure 2:**
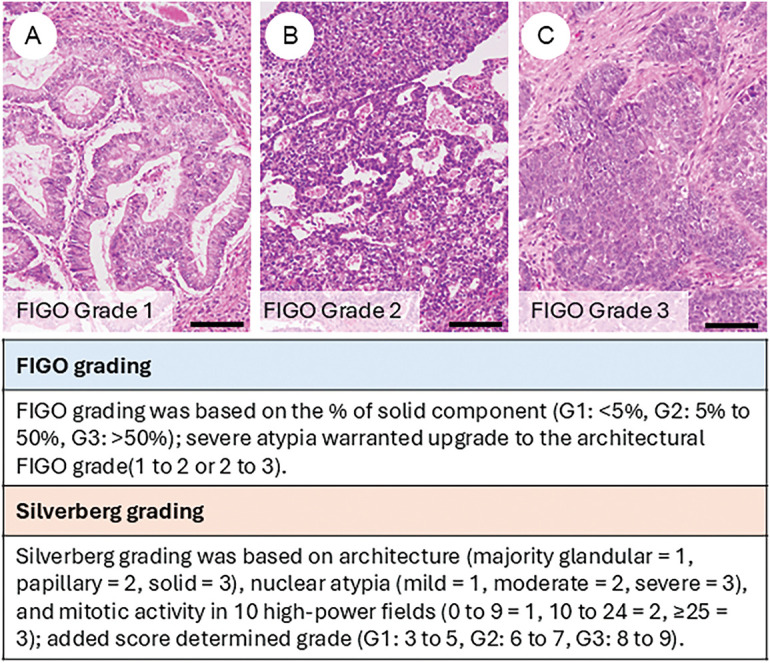
Tumor grading of ovarian endometrioid carcinoma. Representative histological images of FIGO grade 1 (**A**), grade 2 (**B**), and grade 3 (**C**) tumors. The figure also summarizes the criteria for FIGO grading and Silverberg grading systems. The black bar represents 200 μm. All pathology images are original images obtained from the pathology image database of the National Cancer Center Hospital (A–C: ×200)

More recently, Parra-Herran and colleagues supported the Silverberg system for improved prognostic stratification and therapeutic decision-making [50]. Conversely, a study by Soovares et al. supported continued use of FIGO grading [40]. In their analysis of 215 OEC cases, they assessed stage, FIGO grade, clinicopathological factors, and 12 immunohistochemical markers (including progesterone receptor (PR), estrogen receptor (ER), β-catenin, vimentin, ARID1A, HNF1β, p53, p16, MIB-1, E-cadherin, Human epidermal growth factor receptor 2 (HER2), and L1 cell adhesion molecule (L1CAM)) about survival outcomes, identifying both grade and stage as independent prognostic factors. Molecular classification is expected to gain prognostic significance, underscoring the need for integrative validation of stage, molecular subtype, and histologic grade. The prognosis differs between low-grade and high-grade tumors, and in cases of high-grade tumors, distinct treatment strategies—such as additional lymphadenectomy or adjuvant chemotherapy—are applied [51]. Therefore, at least binary grading must be performed with careful consideration and appropriate rigor.

### 5.3 Morphologic and Metaplastic Variants in OEC (Fig. 3)

OEC frequently exhibits diverse morphologic and metaplastic changes, which, although often lacking prognostic significance, are diagnostically relevant and can complicate histopathological interpretation [8,52–54].

**Figure 3:**
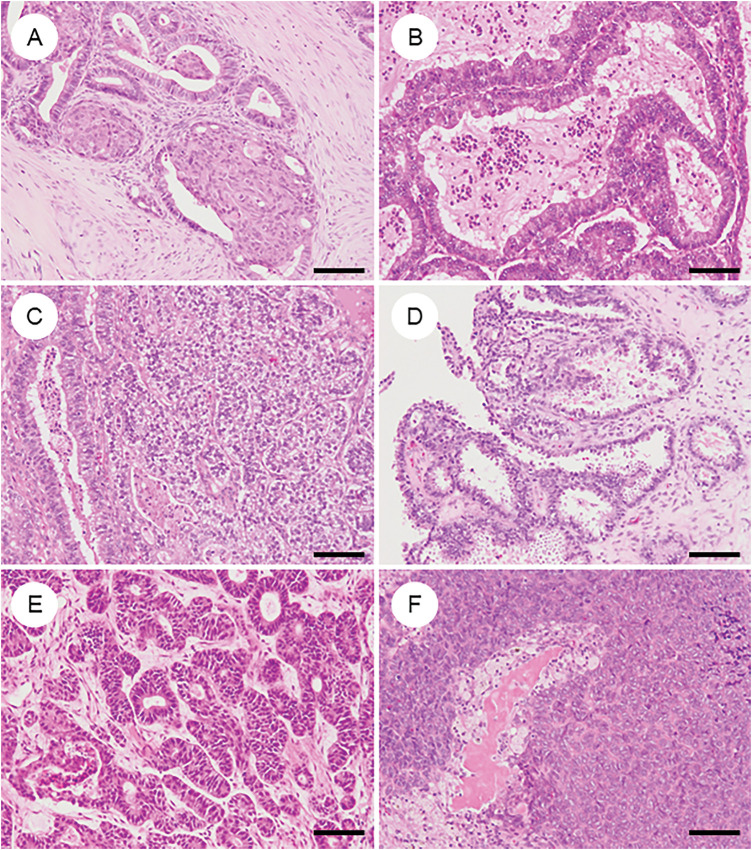
Various metaplastic and morphological changes in ovarian endometrioid carcinoma. (**A**) Morular metaplasia. Bland spindle-shaped cells proliferate within the lumen of tumor glands. (**B**) Mucinous metaplasia. The cytoplasm of tumor cells contains mucin. (**C**) Clear cell or secretory change. Tumor cells have clear cytoplasm and resemble secretory-phase endometrial glands with subnuclear and supranuclear vacuoles. (**D**) Oxyphilic (eosinophilic) cytoplasmic change. The cytoplasm is eosinophilic, with some cells exhibiting a hobnail-like appearance. (**E**) Sex cord-like differentiation. Tumor cells show cord-like or hollow tubule-like architecture. (**F**) Spindle cell morphology and corded and hyalinized pattern of endometrioid carcinoma. Short spindle cells proliferate with associated osteoid formation. The black bar represents 200 μm. All pathology images are original images obtained from the pathology image database of the National Cancer Center Hospital (A–F: ×200)

#### 5.3.1 Squamous and Morular Metaplasia

Squamous or morular metaplasia is present in approximately 50% of OECs [41]. Morular metaplasia (Fig. 3A) consists of clusters of mildly atypical spindle to oval cells forming globular or syncytial structures with eosinophilic cytoplasm and intranuclear inclusions. These lesions can mimic cribriform or solid architecture and may contain central necrosis, potentially impacting tumor grading and differential diagnosis. Morulae display an atypical immunophenotype—positive for caudal-type homeobox protein 2 (CDX2), special AT-rich sequence-binding protein 2 (SATB2), β-catenin, p16, and CD10, but negative for ER, PR, p63, and p40—differentiating them from true squamous metaplasia and correlating strongly with *CTNNB1* mutations [55–57]. Squamous metaplasia, by contrast, shows definitive squamous features, including keratinization and intercellular bridges, and can occasionally progress to squamous carcinoma.

#### 5.3.2 Clear Cell and Secretory Changes

Clear cell changes (Fig. 3C) may be seen in glandular or squamous areas and reflect intracellular glycogen, lipids, or mucin accumulation [58]. They may mimic secretory endometrium or even clear cell carcinoma [41]. However, true mixed ovarian tumors are now recognized to be exceedingly rare (<1%) [59]. When confirmatory endometrioid features are present without regionally distinct histology of OCCC, these features should be interpreted as clear cell change within OEC. A panel of Napsin A, HNF1Β, and PR can help distinguish CCC from OEC with clear cell change, achieving high diagnostic accuracy [60].

#### 5.3.3 Oncocytic Change

Oxyphilic/oncocytic change (Fig. 3D) may occur, with abundant eosinophilic cytoplasm [61], and should not be confused with CCC.

#### 5.3.4 Mucinous Differentiation

Mucinous differentiation (Fig. 3B), often of endocervical type, is common and supports endometrioid lineage, but it may mimic mucinous carcinoma when extensive [52]. Up to 20% of mucinous carcinomas have been reclassified as OEC based on updated morphologic and immunophenotypic criteria [62]. ER, PR, paired box 8 (PAX8), vimentin, and CA125 positivity support OEC, whereas mucinous tumors show opposite staining profiles. PR and vimentin negativity are helpful in predicting mucinous tumors with high sensitivity and specificity [52].

#### 5.3.5 Sex Cord-Like Differentiation

Sex cord-like differentiation (Fig. 3E), including Sertoliform patterns, can mimic sex cord-stromal tumors [63,64]. When predominant, diagnosis relies on coexisting conventional OEC components, endometriosis, or metaplastic elements. Epithelial membrane antigen (EMA), CK7, BerEP4, and PAX8 positivity, along with negativity for inhibin, steroidogenic factor 1 (SF-1), calretinin, and forkhead box L2 (FOXL2), are helpful in distinguishing OEC [8,65].

#### 5.3.6 Endometrioid Carcinoma with Spindle Cells and Corded and Hyalinized Endometrioid Carcinoma

These variants are uncommon and more frequent in the ovary and uterine corpus [66–69]. Both exhibit low-grade endometrioid carcinoma components alongside bland spindle or corded cells within the hyalinized stroma, occasionally containing osteoid, bone, or cartilage (Fig. 3F). These tumors can mimic carcinosarcoma or sex cord-stromal tumors. Immunohistochemically, they show attenuated expression of cytokeratins, PAX8, and hormone receptors; notably, aberrant nuclear β-catenin and CDX2 expression are seen in some cases. Grading is recommended based on the typical endometrioid carcinoma component when present; otherwise, they may be classified as ungradable or grade 3 [66–69].

#### 5.3.7 Other Metaplastic or Unusual Morphological Changes

Ciliated differentiation is occasionally observed without clinical significance [70]. Pilomatrix-like differentiation has been described in both endometrial and ovarian OEC, with high-grade areas resembling cutaneous pilomatrix carcinoma [71–73]. These rare cases may harbor *CTNNB1* mutations and demonstrate aggressive behavior despite arising in otherwise low-grade tumors [72].

Recognizing these morphologic variants is essential for accurate diagnosis, avoiding misclassification with other histotypes, and appropriate grading, especially in the context of evolving molecular taxonomy.

## 6 Immunohistochemical Phenotype of OEC (Fig. 4)

OEC, it is essential to be aware of typical immunoprofiles while also recognizing the potential for aberrant marker expression, so as not to rely overly on immunohistochemistry (IHC) alone. Classically, OECs are positive for ER, PR, CK7, PAX8, and CA125, with focal expression of vimentin [8,41,74]. However, some cases may lack multiple of these markers. Conversely, markers typically associated with non-gynecologic primaries—such as CK20, CDX2, SATB2, thyroid transcription factor-1 (TTF1), Napsin A, GATA binding protein 3 (GATA3), and Wilms tumor 1 (WT1)—can occasionally show focal positivity in OECs [75–80]. WT1, although commonly used as a marker of serous tumors, is reported to be positive in 14% of OECs, particularly those of low grade [8,62]. The p53 staining pattern is usually wild type in grade 1 and 2 tumors. In contrast, a mutant-type pattern (diffuse strong or null) may be seen in a subset of grade 3 tumors, occasionally localized to higher-grade areas—an important clue in distinguishing from high-grade serous carcinoma [62]. Napsin A is generally negative but may be positive in the foci with clear cell change [60]. p16 typically exhibits a mosaic pattern rather than block-type positivity [81]. Genetically correlated markers, such as nuclear β-catenin (reflecting *CTNNB1* mutation) and loss of phosphatase and tensin homolog (PTEN) or ARID1A expression, are also commonly encountered [55,82–85].

**Figure 4:**
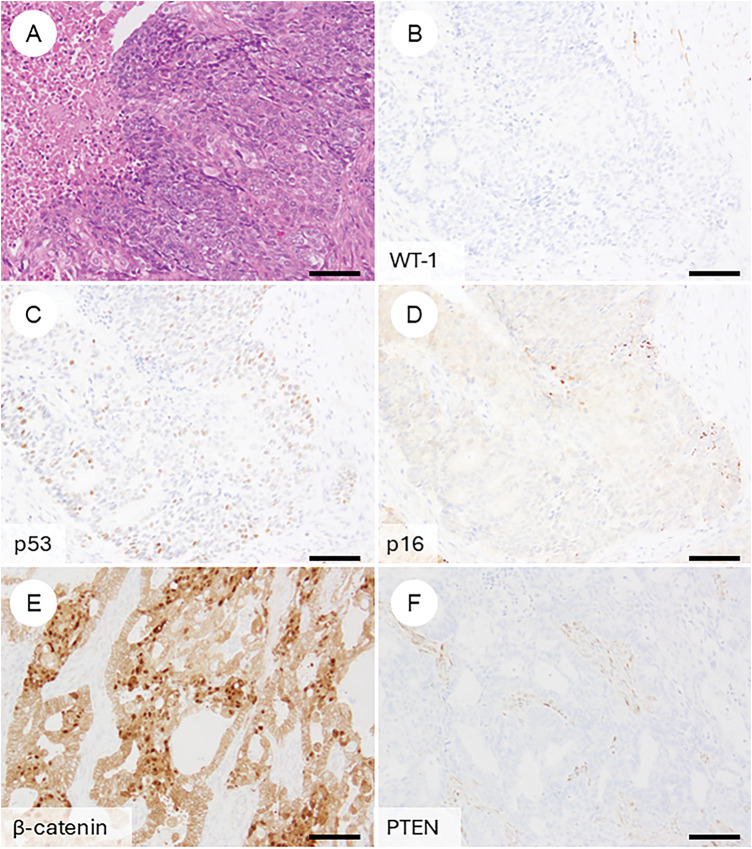

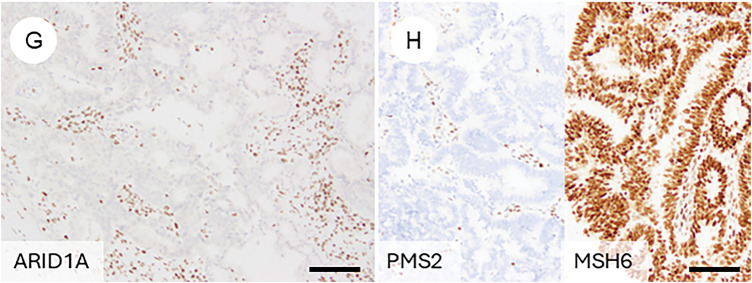
Immunophenotypic features of ovarian endometrioid carcinoma. (**A**) A FIGO grade 3 ovarian endometrioid carcinoma exhibiting cytological atypia that mimics high-grade serous carcinoma. (**B**) The tumor is negative for WT-1. (**C**) p53 shows a wild-type (non-aberrant) staining pattern. (**D**) p16 demonstrates weak, mosaic positivity. Immunohistochemical findings reflecting underlying genetic alterations frequently observed in ovarian endometrioid carcinoma include: (**E**) nuclear accumulation of β-catenin, (**F**) loss of PTEN expression, (**G**) loss of ARID1A expression, and (**H**) mismatch repair deficiency, such as loss of PMS2 with retention of MSH6. The black bar represents 200 μm. All pathology images are original images obtained from the pathology image database of the National Cancer Center Hospital (A–H: ×200)

Neuroendocrine markers, such as chromogranin A and synaptophysin, can be abnormally positive, regardless of tumor grade, even in the absence of neuroendocrine morphology [86]. These findings represent aberrant IHC expression and should not be misinterpreted as evidence of neuroendocrine carcinoma.

Mismatch repair (MMR) deficiency or microsatellite instability (MSI) is present in approximately 12% of OECs, most frequently involving loss of MutL homolog 1 (MLH1) and PMS1 Homolog 2 (PMS2), with *MLH1* promoter methylation accounting for about 70% of such cases [10,87–89]. Approximately 3% are associated with Lynch syndrome [89]. In Western countries, routine MMR IHC is recommended for newly diagnosed OECs (and clear cell carcinomas) to facilitate Lynch syndrome screening [90–92].

Recent findings highlight SRY-box transcription factor 17 (SOX17) as a lineage marker frequently expressed in non-mucinous ovarian carcinomas [93–95], while trichorhinophalangeal syndrome type I (TRPS1)—initially identified as a breast cancer marker—has also been found to be commonly positive in OECs and endometrial carcinomas, necessitating cautious interpretation in differential diagnoses [96].

## 7 Various Differentiations and Related Histological Types

### 7.1 Dedifferentiated and Undifferentiated Carcinoma (Fig. 5A–D)

In rare cases, OEC may contain undifferentiated carcinoma components, a condition referred to as dedifferentiated carcinoma [7,97]. There are also instances where the tumor is composed solely of undifferentiated carcinoma [7,97]. Histologically, undifferentiated components are characterized by diffuse proliferation of medium to large monotonous tumor cells with minimal cohesion, often accompanied by necrosis and numerous mitotic figures [97–99]. Some cases also exhibit marked nuclear pleomorphism, focal keratinization, rhabdoid-like cells, or myxoid stroma [98,99]. Immunohistochemically, undifferentiated carcinoma typically shows focal positivity for cytokeratin and EMA but is negative for ER, PR, and PAX8 [100,101]. Loss of E-cadherin expression is another hallmark feature. Additionally, alterations in the SWI/SNF chromatin remodeling complex are commonly observed and are generally mutually exclusive—these include loss of SWI/SNF related, matrix associated, actin dependent regulator of chromatin, subfamily a, member 4 (SMARCA4), SWI/SNF related, matrix associated, actin dependent regulator of chromatin, subfamily B, member 1 (SMARCB1), or co-inactivation of ARID1A and ARID1B [98,101]. Mismatch repair deficiency is frequently detected in these tumors [98,99,101]. Diagnosis is straightforward when a differentiated carcinoma component is present. However, cases consisting solely of undifferentiated components can be challenging. In such instances, awareness of the potential diagnosis and confirmation of the characteristic immunophenotype are essential for accurate identification and appropriate clinical management.

**Figure 5:**
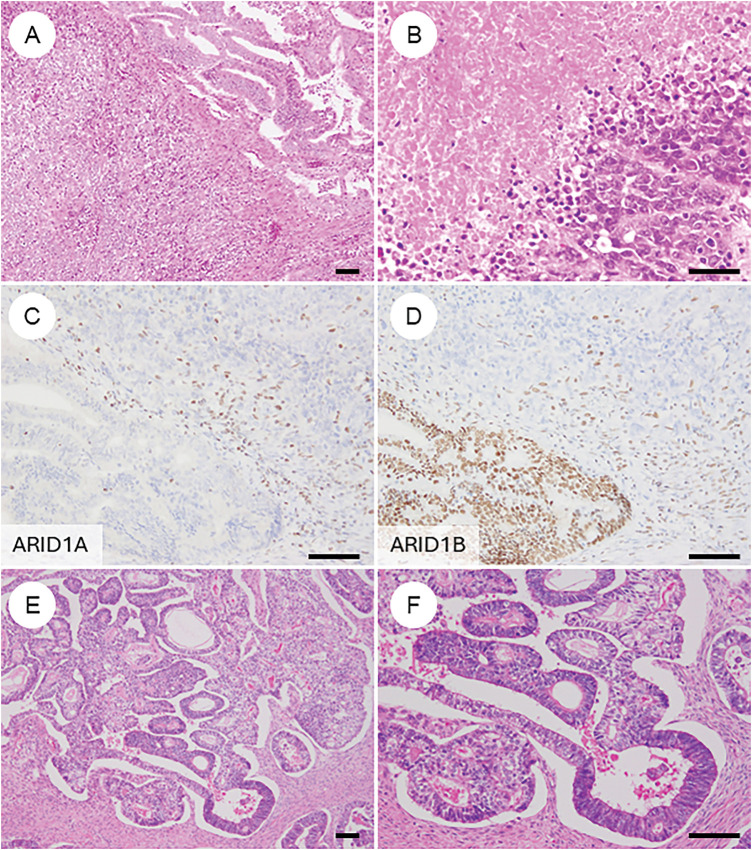

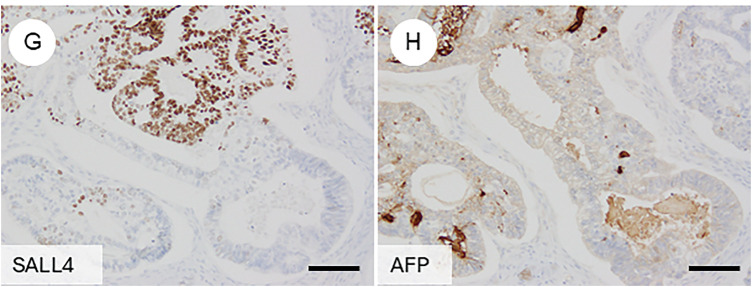
Ovarian endometrioid carcinoma with diverse types of differentiation. (**A**) Ovarian endometrioid carcinoma showing dedifferentiation with an undifferentiated carcinoma component (lower left). (**B**) Undifferentiated carcinoma component with extensive necrosis. (**C**, **D**) Immunohistochemically, the undifferentiated component shows concurrent loss of ARID1A and ARID1B expression. (**E**) In some cases, ovarian endometrioid carcinoma may exhibit a somatically derived yolk sac tumor component. (**F**) The lower right area shows conventional endometrioid carcinoma, while the upper left area shows glandular structures with clear cytoplasm. (**G**, **H**) The yolk sac tumor component is positive for SALL4 and AFP. The black bar represents 200 μm. All pathology images are original images obtained from the pathology image database of the National Cancer Center Hospital (A, E: ×40; B–D, F–H: ×200)

### 7.2 Neuroendocrine Carcinoma

Primary neuroendocrine carcinoma of the ovary is exceedingly rare [7,102], though a few case reports have suggested a stepwise tumorigenesis involving endometriosis, endometrioid carcinoma, and neuroendocrine differentiation [103]. More importantly, aberrant expressions of neuroendocrine markers such as chromogranin A and synaptophysin are occasionally observed in ovarian endometrioid carcinoma [86]. In the absence of corresponding morphological features, a diagnosis of neuroendocrine carcinoma should be avoided.

### 7.3 Somatically Derived Yolk Sac Tumor (Fig. 5E–H)

Ovarian yolk sac tumors (YSTs) typically occur in children and young adults, with cases in patients over 40 years of age being exceedingly rare [7]. In older patients, such tumors are often associated with somatic epithelial neoplasms, most commonly endometrioid carcinoma, and are referred to as somatically derived YSTs [104]. These tumors are thought to arise through aberrant differentiation (trans-differentiation) from epithelial carcinomas [105]. In rare instances, other germ cell tumor components, such as teratomas, may coexist [105]. Genomic analyses of the epithelial and germ cell components have shown nearly identical mutational profiles, with driver mutations corresponding to those typically found in epithelial tumors [106–108].

In contrast, no mutations typically seen in post-pubertal germ cell tumors or gestational trophoblastic neoplasms were detected, and fluorescence *in situ* hybridization (FISH) for i (12p)—a hallmark of germ cell tumors—was negative [106]. These findings suggest that the YST and choriocarcinomatous components are of somatic origin rather than true germ cell derivation. For diagnosis, recognition of the characteristic glandular morphology (the glandular variant being most common) and immunophenotypic expression of markers such as Sal-like protein 4 (SALL4), alpha-fetoprotein (AFP), and glypican-3 is critical for identifying the yolk sac tumor component [109].

## 8 Diagnostic Challenges

### 8.1 Synchronous Endometrioid Carcinoma of the Ovary and Endometrium

Synchronous endometrioid carcinoma of the ovary and endometrium (SEOC) is identified in approximately 15%–20% of OEC cases and about 5% of endometrial carcinoma cases [3]. Accurate distinction between dual primary tumors and metastasis is critical for staging and optimal therapeutic decision-making, aligned with modern classification systems and treatment protocols. Pathologists have traditionally relied on histopathological features—such as tumor size, histologic type, grade, lymphovascular space invasion, and precursor lesions like atypical hyperplasia or endometriosis—to distinguish between independent primaries and metastatic disease [41].

However, advances in genomic profiling have revealed that endometrial and ovarian tumors often share identical molecular alterations in sporadic cases, suggesting a clonal relationship [110–113]. These findings support that most SEOCs represent metastasis from a uterine primary to the ovary, even when morphological criteria imply dual primaries. Notably, studies involving patients with Lynch syndrome have demonstrated that, in some cases, the uterine and ovarian tumors are not clonally related, indicating true independent tumorigenesis. Recent methylation profiling has shown differences in DNA methylation patterns between the endometrial and ovarian tumors, even in clonally related sporadic SEOC cases [114]. This suggests that the ovarian tumor microenvironment may induce epigenetic modifications in metastatic endometrial carcinoma [114].

Despite increasing molecular evidence supporting a metastatic origin for most SEOCs, it is well established that these tumors tend to have favorable clinical outcomes. To avoid overtreatment, the 2023 FIGO staging system for endometrial carcinoma introduced a new substage, IA3, which applies to patients with synchronous, low-grade (G1/G2) endometrioid carcinomas confined to both the endometrium and ovary with superficial myometrial invasion ≤50%, no or focal lymphovascular involvement (LVSI), and no further metastases [115]. This acknowledges the typically indolent clinical course of such cases. In cases where histological subtypes are identical in both sites and a definitive distinction between primary and metastatic lesions is not possible, maintaining a diagnosis of SEOC is acceptable in daily practice. Pathologists should communicate the diagnostic uncertainty and its potential implications for treatment decision-making in such instances [51]. This approach is consistent with ESMO guidelines and is supported by level IV evidence and a grade C recommendation.

Importantly, synchronous tumors show a higher prevalence of MMRd and MSI-H phenotypes (28%) and Lynch syndrome (7%) compared to solitary OEC, underscoring the need for close coordination with clinicians for appropriate germline testing and family counseling [89].

### 8.2 Metastatic Carcinoma, Colorectal Cancer

Due to morphological similarities, OEC can occasionally be challenging to distinguish from metastatic colorectal adenocarcinoma involving the ovary [8]. In most cases, accurate diagnosis can be achieved by integrating clinical information and identifying supportive histological features characteristic of OEC. However, immunohistochemistry becomes a valuable diagnostic tool in specific contexts, such as small biopsy samples or limited clinical data. Immunoprofiles typically supportive of OEC include positivity for PAX8, ER, PR, and CK7. In contrast, colorectal adenocarcinomas typically exhibit diffuse expressions of CK20, CDX2, and SATB2 [116]. These markers are especially helpful when evaluating suspected gastrointestinal metastasis to the ovary or when diagnosing OEC arising from deep infiltrating endometriosis on gastrointestinal biopsy specimens. Nevertheless, caution must be exercised when interpreting immunohistochemical results, particularly in cases with atypical expression patterns. Diagnostic accuracy requires correlation with morphology, clinical context, and awareness of exceptions to typical immunoprofiles.

### 8.3 Endocervical Adenocarcinoma, HPV-Associated

Cervical adenocarcinoma can occasionally metastasize to the ovary [117]. Due to its morphological resemblance—particularly in HPV-associated usual-type endocervical adenocarcinoma—to endometrioid carcinoma of the lower uterine segment, distinguishing ovarian metastases from primary ovarian endometrioid carcinoma can be challenging [118]. However, with appropriate clinical information, identification of a cervical lesion, and confirmation of block-type p16 immunoreactivity as a surrogate marker for HPV-related tumors [81,118], accurate differentiation is usually achievable. The uterine cervix can harbor HPV-independent adenocarcinomas—most notably, gastric-type adenocarcinoma, which characteristically contains intracytoplasmic mucin and exhibits a gastrointestinal-type immunophenotype distinct from that of endometrioid carcinoma. These tumors are typically negative for hormone receptors such as ER and PR, which are usually positive in endometrioid carcinoma [7]. Therefore, differentiation from endometrioid carcinoma is generally not problematic.

### 8.4 Other Primary Ovarian Carcinomas Not Mentioned above

#### 8.4.1 High-Grade Serous Carcinoma (Fig. 6A and B)

Some HGSCs are frequently misclassified as OEC, representing a critical diagnostic pitfall in daily practice [13]. A subset of HGSCs may display histologic features mimicking OEC, such as smooth luminal, fused glandular structures, solid growth, and occasional squamous differentiation. These features, known as the SET (solid, endometrioid, transitional) pattern, have been associated with Breast Cancer gene 1/2 (*BRCA1/2*) mutations and homologous recombination deficiency (HRD). Distinguishing such cases requires careful integration of both morphological and immunohistochemical findings. Supporting evidence for OEC includes the presence of “confirmatory endometrioid features” and a wild-type p53 immunostaining pattern, along with WT1 negativity [13,46]. Additional immunohistochemical markers such as MMR proteins, β-catenin, ARID1A, PTEN, and vimentin may also provide supportive clues for accurate classification.

**Figure 6:**
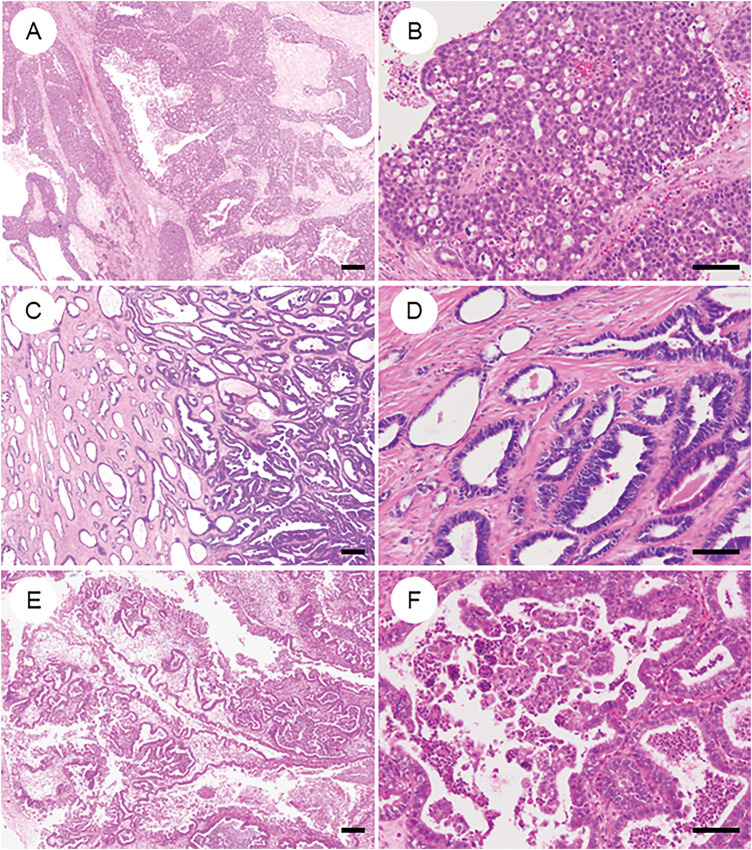
Primary ovarian neoplasms that may be mistaken for ovarian endometrioid carcinoma. (**A**, **B**) High-grade serous carcinoma exhibiting the solid, endometrioid, and transitional (SET) pattern, thereby closely mimicking endometrioid carcinoma. (**C**, **D**) Mesonephric-like adenocarcinoma characterized by prominent tubular and tubulopapillary architecture; historically, many such tumors were misclassified as low-grade endometrioid carcinoma. (**E**, **F**) Seromucinous tumor displaying mixed Müllerian-type epithelial differentiation. Notably, seromucinous carcinoma is considered a subtype of endometrioid carcinoma in the 2020 WHO classification. The black bar represents 200 μm. All pathology images are original images obtained from the pathology image database of the National Cancer Center Hospital (A, C, E: ×40; B, D, F: ×200)

#### 8.4.2 Mesonephric-Like Adenocarcinoma (Fig. 6C and D)

Mesonephric-like adenocarcinoma (MLA) is a rare subtype of endometriosis-associated ovarian neoplasm [119,120] that was previously diagnosed in most cases as low- to intermediate-grade endometrioid carcinoma (G1/2) [8]. Clinically, MLA often presents with recurrence or distant metastasis [119,120]. In addition to its characteristic cytological and architectural features, the lack of metaplastic changes and hormone receptor expression (ER/PR negativity), alongside immunopositivity for GATA3, TTF1, and, more recently, paired box gene 2 (PAX2), helps distinguish MLA from endometrioid carcinoma [121]. Despite its name, the term “MLA” is not considered a true mesonephric tumor of Wolffian origin; instead, it is believed to arise from Müllerian neoplasms that have undergone mesonephric-like differentiation [120,122]. Mixed tumors and endometrioid carcinomas with partial MLA-like morphology and immunophenotype are increasingly recognized, highlighting the need for further case collection and refinement of diagnostic criteria. Notably, *KRAS* mutations are frequently observed in MLA [123,124]. Ongoing clinical trials are investigating the efficacy of targeted therapies against *KRAS*-mutant tumors, drawing attention to this molecular alteration as a potential therapeutic vulnerability [125].

#### 8.4.3 Seromucinous Carcinoma (Fig. 6E and F)

In the 2014 WHO classification, seromucinous carcinoma was described as the malignant counterpart of seromucinous tumors [126]. However, subsequent studies evaluating diagnostic reproducibility, immunophenotype, and molecular profiles concluded that it lacks sufficient distinction as an independent histotype [127]. Consequently, the 2020 WHO classification removed this category, and seromucinous carcinoma is now considered a variant of endometrioid carcinoma [7].

#### 8.4.4 Histopathological Diagnosis Aided by Artificial Intelligence (AI)

In recent years, AI, particularly deep learning (DL)-based approaches, has emerged as a promising tool for assisting histopathological diagnosis [128] of ovarian carcinoma. Farahani et al. developed a DL-based classifier trained on whole-slide images of five major histologic subtypes, including OEC [129]. Using 948 whole-slide images from 485 patients for training, and an independent test set of 60 patients, four convolutional neural network models were developed. The best model achieved a diagnostic concordance of 81.38% (kappa = 0.7378) on the training set and 80.97% (kappa = 0.7547) on the external test set [129]. These results suggest that AI models may enhance diagnostic accuracy when used alongside traditional histopathology. Although still in early clinical adoption, such DL-assisted tools are expected to enhance diagnostic reproducibility and may serve as a valuable adjunct for pathologists facing histologic overlap between OEC and other ovarian tumors.

## 9 Molecular Landscape of OEC

Recent genomic studies have identified several recurrent mutations in OEC. High-frequency alterations include mutations in *CTNNB1* (30%–50%), *PIK3CA* (30%–50%), *KRAS* (25%–40%), *ARID1A* (20%–40%), and *PTEN* (30%–45%) [9,130–132]. Earlier reports suggested a *TP53* mutation frequency exceeding 50%, likely due to misclassified HGSC within OEC cohorts [133]. In strictly defined OEC populations, *TP53* mutations are now observed in approximately 20% of cases, predominantly in high-grade tumors [9,130–132]. A recent whole-exome sequencing study by Hollis et al. on 112 rigorously selected OEC revealed *CTNNB1* and *PIK3CA* mutations in 43% of cases, *ARID1A* in 36%, *PTEN* in 29%, *KRAS* in 26%, and *TP53* in 26%. *SOX8* mutations were also detected in 19% of cases [9]. Notably, DNA polymerase epsilon, catalytic subunit (*POLE*) exonuclease domain mutations were found in 6% and MMR gene alterations in 18%, with considerable co-occurrence between the two [9]. *BRCA1/2* mutations were identified in approximately 10% of high-grade OEC cases [134].

*ARID1A* mutations, frequently seen in both OEC and OCCC, are known to be a hallmark of endometriosis-associated ovarian cancers [27]. Recent evidence suggests that ARID1A loss is not a prognostic marker [135]. A large-scale immunohistochemical analysis conducted by an international team, utilizing the Ovarian Tumor Tissue Analysis consortium, the Canadian COEUR resource, and other collaborating institutions, evaluated 1623 endometriosis-associated ovarian carcinoma cases (1078 OECs and 545 OCCC) [135]. Loss of ARID1A expression, used as a validated surrogate for mutation, was observed in 42% of OCCC and 25% of OECs. Significantly, ARID1A loss did not correlate with clinical outcomes, stage, age, or CD8+ tumor-infiltrating lymphocyte (TIL) levels in either histotype, suggesting that while *ARID1A* mutation is a key molecular event in tumorigenesis, it lacks prognostic significance [135].

Identifying pathogenic variants (PVs) in *BRCA1* and *BRCA2* in epithelial ovarian cancer is crucial for guiding genetic counseling and therapeutic decision-making [136,137]. Accordingly, current American Society of Clinical Oncology guidelines recommend *BRCA1/2* sequencing for all ovarian cancer patients, irrespective of histologic subtype [136]. *BRCA1/2* PVs are most frequently observed in HGSC. Kramer et al. analyzed *BRCA1/2* tumor sequencing results from a centrally reviewed prospective cohort encompassing all histologic subtypes [134]. Among 946 sequenced cases, 125 (13%) harbored *BRCA1/2* PVs, of which 117 occurred in HGSC. Only eight *BRCA1/2* PVs were found in non-HGSC tumors. After re-evaluating histologic classification and pathogenicity, only two out of 20 confirmed high-grade OEC (10%) retained germline *BRCA2* PVs with presumed pathogenic relevance [134]. No clinically meaningful *BRCA1/2* alterations were identified in other histologic subtypes. These findings suggest that *BRCA1/2* mutations may contribute to tumorigenesis in rare cases of high-grade OEC; they are unlikely to be relevant in other subtypes. Although further validation is warranted, the data support a potential shift from universal *BRCA1/2* testing to a histology-driven, selective sequencing approach [51].

Human epidermal growth factor receptor 2 (HER2), located on chromosome 17q12, belongs to the epidermal growth factor receptor family and functions as an upstream activator of the PI3K/AKT signaling pathway, which regulates cell proliferation, survival, and invasion [138]. HER2 overexpression has been implicated in the tumorigenesis of various malignancies and serves as a predictive biomarker for HER2-targeted therapies, including in gynecologic cancers [139]. Several clinical trials are currently evaluating the efficacy of HER2-targeted drugs in ovarian carcinoma, with promising results [139]. However, HER2 gene amplification has been reported in only a small subset (~4%) of OEC. In a recent study by Bui et al., immunohistochemical assessment of HER2 (clone 4B5, Ventana platform) in 48 cases of OEC revealed two cases with a score of 2+ and 12 cases with a score of 1+ [140]. Prior studies have reported HER2 overexpression rates in OEC ranging from 2.1% to 23.1% [140]. Notably, although only 2.1% of cases in Bui’s study demonstrated true overexpression, 29.2% exhibited some degree of HER2 expression (score ≥1+), suggesting a substantial proportion of tumors might be eligible for HER2-targeted antibody-drug conjugate (ADC) therapies [139].

Recent integrative epigenomic and transcriptomic analyses have revealed that epigenetic alterations, including DNA methylation and chromatin remodeling, play a crucial role in histotype-specific tumor biology in early-stage ovarian carcinomas. In a comprehensive study by Swenson et al. [141], differential analyses of DNA methylation and gene expression were conducted on 86 early-stage epithelial ovarian cancer samples that had undergone histotype-based pathological reclassification. Correlations between DNA methylation and gene expression were examined to identify histotype-specific biomarkers. Notably, some candidate biomarkers effectively distinguished clear cell carcinoma, high-grade serous carcinoma, and mucinous carcinoma at the transcriptional level. In contrast, further stratification or potential reclassification for OEC may be warranted in future investigations [141].

## 10 TCGA-Based Molecular Classification in OEC

**Figure 7:**
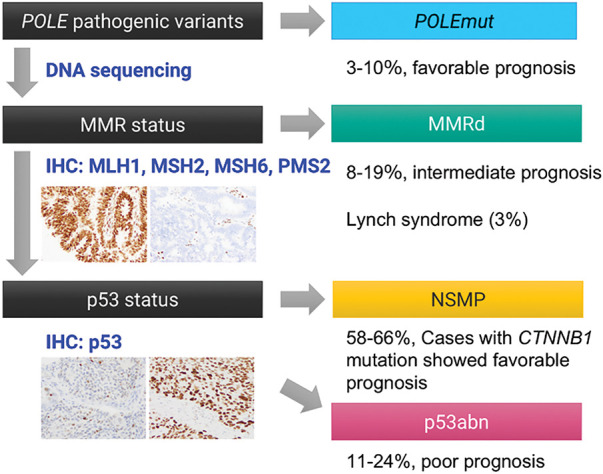
TCGA-surrogate molecular classification of ovarian endometrioid carcinoma. Accumulating evidence suggests that the TCGA-based molecular classification system, originally developed for endometrial carcinoma, is also applicable to ovarian endometrioid carcinoma. Tumors can be stratified into four molecular subtypes—POLEmut, MMRd, NSMP (no specific molecular profile), and p53abn—based on the presence of pathogenic mutations in the POLE exonuclease domain, loss of mismatch repair (MMR) protein expression, and abnormal p53 immunostaining patterns

In 2013, The Cancer Genome Atlas (TCGA) fundamentally reshaped the understanding of endometrial carcinoma, revealing its molecular heterogeneity and classifying it into four prognostically and therapeutically relevant subgroups: (1) *POLE*-ultramutated (*POLE*mut), characterized by excellent prognosis regardless of histologic grade; (2) p53-abnormal (p53abn, or serous-like), associated with poor prognosis; (3) MMRd, with intermediate prognosis; and (4) no specific molecular profile (NSMP), a heterogeneous group lacking defining molecular alterations [142]. This molecular classification is increasingly incorporated into routine diagnostic, prognostic, and therapeutic algorithms for endometrial cancer (Fig. 7) [7,115]. Considering the morphological resemblance of OEC, attempts to implement this molecular classification to OEC seem to be a natural course.

Recent studies applying this TCGA-based classification to OEC have demonstrated a similar distribution into four molecular subtypes: *POLE*mut (3%–10%), MMRd (8%–19%), p53abn (11%–24%), and NSMP (58%–66%) [9,10,39,131,143–145]. Hoang et al. examined 89 OECs and identified *POLE* exonuclease domain mutations in 4.5% of cases, restricted to low-grade tumors. All mutations were somatic missense variants at known hotspots (P286R, V411L). While none of the POLE-mutant patients experienced recurrence, the difference was insignificant. These findings contrast with endometrial cancer, where *POLE* mutations are more often found in high-grade tumors [144]. In 2017, Parra-Herran et al. evaluated 72 OECs using immunohistochemistry and *POLE* sequencing, classifying tumors into four subtypes: *POLE*mut (10%), MMRd (8%), p53abn (24%), and p53 wild-type (58%). Disease-free survival differed significantly by subtype (*p* = 0.003), independent of tumor grade or stage. *POLE*mut and MMRd tumors showed excellent prognosis, while p53abn cases were associated with higher recurrence and mortality [143]. Cybulska et al. performed targeted or whole-genome sequencing on 36 pure OEC cases. They classified tumors as *POLE*mut (3%), MSI-high (19%), copy-number high/serous-like (17%), and copy-number low (61%). *AKT1 (AKT serine/threonine kinase 1)* and erb-b2 receptor tyrosine kinase 2 (*ERBB2*) hotspot mutations were identified in a subset, and MSI-high tumors had uniformly favorable outcomes [131]. Hollis et al. conducted whole-exome sequencing on 112 rigorously reviewed OEC cases. Frequent mutations included *CTNNB1* (43%), *PIK3CA* (43%), *ARID1A* (36%), *PTEN* (29%), *KRAS* (26%), *TP53* (26%), and *SOX8* (19%). *POLE* and MMR gene mutations occurred in 6% and 18% of cases, respectively, with co-occurrence observed. *TP53*-mutant tumors showed greater genomic complexity, advanced stage (48%), incomplete debulking (44%), and poor prognosis. In contrast, *CTNNB1*-mutant tumors (mutually exclusive with *TP53* mutations) were typically in the early stages and completely resected (87%), with excellent outcomes. The authors also identified *WNT, MAPK/RAS*, and *PI3K* pathways as promising therapeutic targets in OEC [9].

Krämer et al. analyzed 511 OECs using TCGA-surrogate classification, which revealed the following distributions: *POLE*mut (3.5%), MMRd (13.7%), p53abn (9.6%), and NSMP (73.2%). Distinct survival outcomes were observed (*p* < 0.001), consistent with endometrial carcinoma. Subtypes remained prognostic even after adjusting for clinical variables, suggesting value in treatment stratification, including for fertility-preserving approaches [10].

Leskela et al. applied the same classification to 166 early-stage OECs: *POLE*mut (5%), MMRd (18%), p53abn (11%), and NSMP (66%). Five cases met the criteria for multiple subtypes. Histological and immunophenotypic features differed by subtype, including expression of ARID1A, β-catenin, ER, Napsin A, and HNF1Β. CD8+ TLSs were increased in *POLE*mut and MMRd groups. These findings support the clinical utility of molecular classification in OEC, with *POLE* and MMR alterations identifying tumors with a favorable prognosis and potential responsiveness to immunotherapy [39].

These accumulating data strongly suggest that TCGA-derived molecular classification provides meaningful prognostic stratification for OEC, enabling more precise risk assessment and potentially guiding tailored therapeutic approaches [9].

## 11 Lynch Syndrome and MMR-Deficiency in OEC

Lynch syndrome (LS) is an autosomal dominant cancer predisposition syndrome caused by germline mutations in one of the DNA mismatch repair genes—*MLH1, PMS2, MSH2, MSH6*, or *EPCAM (Epithelial Cell Adhesion Molecule)* [146,147]. LS is most strongly associated with colorectal and endometrial carcinomas, and universal screening for LS in these malignancies has been adopted in Western countries [90–92]. Although ovarian cancer is less commonly associated with LS, the lifetime risk of ovarian cancer in LS carriers is estimated at 9%–12% [146]. The lifetime risk of developing ovarian cancer in individuals with Lynch syndrome has been reported to be approximately 11.0% (7.4–19.7) for MLH1, 17.4% (11.8–31.2) for MSH2, 10.8% (3.7–38.6) for MSH6, and 3.0% (0.5–43.3) for PMS2 pathogenic variant carriers [148]. Furthermore, up to 7% of patients with non-serous, non-mucinous epithelial ovarian carcinomas may carry germline LS mutations [149]. Since gynecologic malignancies may be the sentinel cancers in LS patients, identifying such cases can provide opportunities for cancer surveillance and preventive interventions for both the patients and their first-degree relatives [149].

A systematic review by Mitric et al. evaluated the prevalence of MMRd, MSI-high, and confirmed LS in ovarian cancer [89]. The systematic review included 55 studies and assessed the sensitivity and specificity of various screening methods, using NGS as a reference. The pooled prevalence was 6% for MMRd (95% CI: 5%–8%), 13% for MSI-high (95% CI: 12%–15%), and 2% for germline-confirmed LS (95% CI: 1%–3%). Notably, 76% of MLH1-deficient tumors showed *MLH1* promoter hypermethylation, indicating a sporadic etiology rather than LS [89]. By histological subtype, OEC showed the highest frequency of MMRd (12%) and MSI-high (12%). In contrast, serous carcinomas had the lowest MMRd frequency (1%) and MSI-high frequency (9%). Non-serous, non-mucinous carcinomas overall had a 9% MMRd. The highest LS mutation prevalence (7%) was observed in cases with synchronous endometrial and ovarian carcinomas, followed by endometrioid (3%) and serous carcinomas (1%). The review also compared the performance of LS screening methods. IHC alone had the highest sensitivity (91.1%), which improved slightly when combined with MSI testing (92.8%). The highest specificity was achieved using IHC combined with MLH1 methylation analysis (92.3%) [89]. Based on these findings, the authors conclude that MMR IHC and germline testing for LS should be considered in non-serous, non-mucinous epithelial ovarian cancers—particularly in the endometrioid subtype—to facilitate early diagnosis, appropriate management, and familial cancer prevention [89].

## 12 Therapeutic Implications Based on Molecular Alterations

Initially developed for endometrial carcinoma, molecular classification has been effectively applied to OEC, offering significant insights for precision medicine [9,10,39,131,143–145]. The MMRd subtype, characterized by high microsatellite instability, often responds well to immune checkpoint inhibitors such as pembrolizumab [39]. This therapeutic approach has shown efficacy in MMRd endometrial cancers and is being explored for OECs with similar molecular features [150]. Recent advances in immunotherapy have demonstrated that MMRd tumors, including a subset of OEC, exhibit a favorable immunogenic profile due to their high tumor mutational burden and consequent neo-antigen production. These features stimulate increased infiltration of cytotoxic CD8^+^ TILs and upregulation of immune checkpoint pathways, particularly the PD-1/PD-L1 pathway. A recent multi-cohort study evaluating nivolumab in MMRd tumors, including ovarian cancers, showed durable responses and survival benefit, underscoring the therapeutic potential of immune checkpoint inhibitors in this context [151]. In MMRd OECs, the presence of abundant CD8^+^ TILs, elevated PD-L1 expression, and interferon-γ-related gene signatures has been associated with enhanced responsiveness to ICIs such as nivolumab and dostarlimab. These features may serve as predictive biomarkers, aiding in the selection of patients for ICI-based therapies. Taken together, accumulating evidence supports the integration of immune checkpoint blockade in the management of MMRd OEC, especially for patients with advanced or recurrent disease.

The p53abn subtype is associated with a poorer prognosis and may benefit from tailored treatment strategies. While platinum-based chemotherapy remains standard, ongoing research is investigating the potential of poly(ADP-ribose) polymerase (PARP) inhibitors, especially in tumors with concurrent *BRCA* mutations or homologous recombination deficiencies. High-grade OEC with p53 abnormalities may benefit from these therapeutics. In contrast, patients with *POLE*mut tumors generally have an excellent prognosis. This favorable outcome suggests the possibility of de-escalating therapy to minimize treatment-related morbidity without compromising efficacy. Beyond these classifications, targeted therapies are being developed based on specific molecular alterations. Mutations in the *PIK3CA/AKT/mTOR, WNT, MAPK/RAS* pathway present opportunities for targeted inhibition [9,152], and ARID1A-deficient tumors may be susceptible to synthetic lethality strategies [153].

The integration of liquid biopsy approaches [154], particularly ctDNA-based assays, holds significant promise for the non-invasive monitoring of OEC. While most current evidence originates from studies in high-grade serous carcinoma, the application of ctDNA for detecting minimal residual disease, monitoring recurrence, and assessing therapeutic response in OEC represents a compelling direction for future research [155,156].

## 13 Future Directions and Research Gaps

A growing body of research highlights the clinical utility of molecular classification in OEC [9,10,39,131,143–145], but significant gaps remain in its prospective validation. Future directions must integrate molecular subtyping—*POLE*mut, MMRd, p53abn, and NSMP, including alterations in *CTNNB1* and/or *KRAS*—into prospective clinical trials to evaluate their impact on prognosis and treatment stratification [9,10,39,51,131,143–145].

In parallel, comprehensive multi-omics approaches—including whole-exome sequencing, transcriptomics, epigenomics, and proteomics—offer promise in refining the molecular landscape of OEC beyond current classifications [157,158]. Such analyses can identify novel molecular subgroups, druggable targets, and resistance mechanisms, thereby facilitating personalized therapeutic strategies. Integrating these data into routine diagnostics could transform prognostication and clinical decision-making [128].

Emerging single-cell technologies, including scRNA-seq and single-cell multi-omics, are expected to offer unprecedented resolution in characterizing the cellular heterogeneity and evolutionary dynamics of OEC. Although such approaches have been extensively applied in high-grade serous carcinoma [159–161], their adoption in OEC remains limited. Future studies utilizing single-cell approaches will likely contribute to a more precise understanding of tumor biology, microenvironmental interactions, and therapeutic vulnerabilities in this histological subtype.

Moreover, recent investigations into the tumor microenvironment (TME) and immune contexture of OEC reveal subtype-specific immune landscapes. For instance, MMRd and *POLE*mut tumors exhibit high tumor-infiltrating lymphocytes and inflammatory signatures, potentially predicting response to immune checkpoint inhibitors. In contrast, p53abn and NSMP tumors may exhibit an immunosuppressive or “cold” TME, necessitating combination therapies [162]. Future translational studies are needed to characterize these immunophenotypes and integrate TME markers into clinical algorithms for immunotherapy selection.

Although our review primarily focuses on established histologic features and molecular pathways of OEC, several emerging areas warrant further investigation. These include tumor microenvironment heterogeneity, such as intratumoral microbial diversity and its potential influence on tumor progression and immunotherapeutic response [163]. Additionally, the integration of network pharmacology and multi-omics technologies offers a promising avenue for uncovering novel therapeutic targets and biomarkers in OEC. Future studies specifically designed to address these gaps may substantially advance our understanding of OEC pathogenesis and support the development of tailored therapeutic strategies.

## 14 Conclusion

Recent advances in the molecular pathology of OEC have revealed distinct biological characteristics that set it apart from other ovarian cancer subtypes, underscoring the need for precision medicine approaches. Integrating histopathological assessment with molecular classification is essential for accurate diagnosis, prognostication, and treatment planning. Universal screening for Lynch syndrome in OEC is clinically important, given its implications for patients and their families. Furthermore, identifying actionable molecular alterations highlights the potential for targeted therapies. Continued efforts to implement molecularly informed strategies in routine practice will enhance personalized care and improve outcomes for patients with OEC.

## Data Availability

Not applicable.
